# Dental Therapeutics

**Published:** 1876-07

**Authors:** S. H. Henkel


### ARTICLE II.

### *Dental Therapeutics.*

BY S. H. HENKEL, D. D. S.

The word Therapeutic is derived from the Greek word *Therapeuo*, and means, " I wait on the sick," " I alleviate or assuage."

Dental Therapeutics is that branch of medicine which has for its object the treatment of diseases of the teeth and associate parts, or which considers the application of the remedies employed for the prevention and cure of the various diseases which human teeth fall heir to in these degenerate days.

To give or discuss the many remedies which our pharmacy gives, would be an endless task; yet in order to intelligently use and apply even those most frequently demanded, we need much information, and our storehouse of learning will often be exhausted, and we be left to grope our way in the dark. We contend that we should have general information, sufficient at least, to give a practical reason for any application chemically made to a diseased tissue. How can we intelligently make such an application if we are ignorant of chemistry? How can we correctly apply a chemical to diseased parts if we are not acquainted with pathology in a sufficient degree, to tell by what the disease is caused, or to foretell its unaided results. How can we give a pathological reason if we do not understand physiology? How can we draw physiological conclusions unless we have studied anatomy? How can we learn the anatomical structure of man in all its complicated organisms, unless we have devoted time and study to all? For none of us have been born great enough to comprehend even the simplest parts of any of the branches we have passed over, or to draw one correct scientific conclusion from the application of any remedy used to allay pain and give ease to suffering humanity.

First, we wish to notice Heat and Cold therapeutically applied. The terms Heat and Cold therapeutically used, do not refer to any particular degree of temperature, but extend from 100° F. to 180° F. for hot, and from 42° to 48° F. for cold. We all know that the action of relative heat and cold upon inanimate things is to produce correspondence of temperature; but animate organisms resist such an equalization, more or less to the extent of their power of vital resistance, and if heat is continuously applied beyond their power of natural resistance, vitality will be destroyed by coagulating the albumen of the blood,—and again on the other hand if the temperature of the living body be reduced to 32° F. by withdrawal of heat, or applications of cold, vitality is destroyed by congealing the water of the blood. Heat which is sufficient to destroy vitality, is according to its intensity proportionately stimulating, and if persistently applied, causes inflammation; yet if applied from a correct therapeutical stand point, becomes a remedial agent of great value, and can be classed among the list of stimulants, for it causes an increased flow of blood to the part to which it is applied, and an increased flow of blood to any special point causes that part to become stimulated, and of course built up, or greatly increased in materials such as the blood carries to build up our wasting structure.

Odontalgia, caused by want of circulation of blood to teeth and associate parts, will be greatly relieved by application of heat. We again assist nature to break down diseased structure of muscular animal tissue, by using hot poultices, thereby hastening a discharge of pus, when the inflammation has progressed too far to have the heat act as a resolvent; yet, if applied sufficiently early in the inflammation, it acts promptly as a stimulant, and brings about resolution. Heat applied in anæmic cases, in a limited form, acts as a stimulant sufficient to increase circulation, to nicety of weakened parts, and is very beneficial in restoring healthy circulation. Heat being stimulating, cold would naturally be considered sedative, or depressing. This is the case where cold is used in insufficient amount to destroy vitality, for if persisted in, it will congeal the water of the blood, thereby checking circulation in parts to which it is used. In many cases where inflammation has set in, cold comes in as a therapeutic agent, as an astringent, and gives relief by congesting and toning up weakened blood vessels, thereby lessening the amount of blood flowing into the capilliaries, and if applied in sufficient quantity, and continuously, allows the overworked capilliaries rest sufficient to allow them to return to their normal size, and to withstand reaction ; or in other words, cold becomes a tonic as well as an astringent. "Inflammation we all know is a condition of things which is induced by long continued peculiarly exciting influences—vitality is called upon for maintenance of systemic or local integrity, to such an extent as no longer to be able to respond without aid." Nature can again be aided by application of cold, thereby astringing, sedating and relieving the parts of the great demand made upon the already weakened structure—giving them rest sufficient to regain self-support. If cold or cooling applications are resorted to, care must be exercised ; first, that their continuance is uninterrupted ; second, that they are employed for sufficient length of time, to allow the parts to regain that degree of tenacity which should enable them to preserve their integrity ; third, that the intensity of cold shall not be such as to destroy vitality. If cool applications afford comfort, and gradual restoration is observed to progress, it will be sufficient reason to continue our treatment ; but, if on the contrary, pain supervenes after a period of cessation, and increases in violence, warm applications may often be resorted to with much benefit, to such an extent as is sedative and sufficiently stimulating, yet not hot enough to become irritating.

We will now leave heat and cold and take up a chemical combination, which we have found a very efficient agent in alveolar abscess.

*Carbolate of Iodine.*—Having used carbolic acid and iodine equally combined, for over six years in chronic abscess, and to such great satisfaction, we can but speak favorably and recommend its use, but with great care and without slovenness; for to succeed in curing diseased tissue successfully, we can hardly be too cleanly or careful. When we find abscess or sac at the fang of a tooth, our first step is to carefully remove all decomposed tooth substance, after which, apply on floss silk carbolic acid and iodine, stopping external opening in the tooth with Hill's Stopping, and discharge our patient with instruction to return in 7 or 8 days if no pain, but if pain follows, to come at once. Generally two or three applications are sufficient to close fistulous opening and destroy sac, after which we proceed to fill permanently. Carbolic acid is a caustic which only affects a superficial layer of the surface to which it is applied, and at the same time it possesses the power of correcting or changing acrid pus to healthy pus, which we generally find issuing from fistulous opening from sac of diseased teeth. Iodine causes or excites new action in walls of cavity or sac, and possesses the power of obliterating it in many cases by adhesive inflammation, or restoring its secreting surface to a healthy condition, and it operates as a general excitant, of the vital actions, especially of the absorbent or glandular system. The condition of an abscess tooth, is a sac formed at the fang, with a well defined wall, protecting or dividing diseased from healthy tissue; internal portion of sac possessing chronic property of forming unhealthy pus, which is constantly or at intermitting periods, discharged from fistulous opening, or through opening in the tooth. We claim that carbolic acid introduced into this sac destroys animalculæ contained in this acrid pus; whilst the iodine assisted by the escharotic action of the carbolic acid causes adhesive inflammation. The iodine having produced adhesive inflammation, changes or breaks up the walls of sac, the inflammation extending beyond diseased tissue, implicates or inflames surrounding healthy tissue, which makes all as one common inflamed structure. Then the recuperative forces restore all to health, after which the tooth can be filled fang and crown.

We will now notice another substance which has raised quite a furore, but already is its meteoric brightness growing dim under the powerful lens of scientific investigation, and that is—salicylic acid. It is claimed for this new chemical that it possesses properties of carbolic acid.

We as dental therapeuticians have for some years, (as said before,) used carbolic acid in many cases ,and many are our successes in arresting decomposing pulp tissue, yet with all our success we have the unpleasant odor and poisonous properties to guard against, which make it not at all attractive. All who are endeavoring to advance, must hail with delight the introduction of a substance that possesses the antiseptic properties of carbolic acid, minus the poison and odor. This is claimed for salicylic acid. What is salicylic acid? In order to fully understand this new chemical, it will be necessary to step into chemistry, for unless we are able to give the chemistry, we will not be competent to use it with intelligence therapeutically. We find by investigation that this new chemical is represented as follows:

| Salicin. | | Water. | | Glucose. | | Saligenin. |
|---|---|---|---|---|---|---|
| $C_{12}H_{18}O_7$ | $+$ | $H_2O$ | $=$ | $C_6H_{12}O_6$ | $+$ | $C_7H_8O_2$ |

When saligenin is submitted to the action of cromate of potash and sulphuric acid, two atoms of hydrogen are oxydized out of it, and becomes water and $C_7H_8O_2$ leaves $C_7H_6O_2$, a thin colorless and very fragrant oil, heavier than water, but soluble in it. This is called Salicilol.

When this salicilol is oxydized by chromic acid, it takes up an atom of oxygen, and $C_7H_6O_2$ becomes $C_7H_6O_3$ which is salicylic acid. To obtain salicylic acid, carbolic acid is exposed to the action of carbonic acid and sodium to loosen the bands between C. H. & O predisposing them to a new combination as follows:

| Carbolic Acid. | | Carbonic Acid. | | Salicylic Acid. |
|---|---|---|---|---|
| $C_6H_6O$ | + | $CO_2$ | = | $C_7H_6O_3$ |

So chemically this new chemical seems really to be carbolic acid, minus the carbonic acid $CO_2$—its escharotic power, and when this new remedy came to light chemical therapeuticians claimed that for all cases where carbolic acid was used, salicylic acid was far superior. We cheerfully and expectantly gave it a trial, and have come to the conclusion, that in alveolar abscess it does not act, or answer in case of carbolic acid, as was claimed for it; for the carbonic acid, which is taken from the carbolic acid, in producing salicylic acid, seems to be the odor as well as the active escharotic principal contained in carbolic acid. Salicylic acid is antiseptic, but not escharotic. We find where there is a considerable amount of decomposed animal tissue that salicylic acid checks farther decomposition, yet we cannot feel justifiable in substituting salicylic acid for carbolic acid.

This, Mr. President and gentlemen, is our imperfect report on dental therapeutics, which we ask you to consider, and if you see anything therein to criticise, we will be gratified to hear and learn your news.

